# The New and the Old

**Published:** 1900-11

**Authors:** 


### Editorial.

### THE NEW AND THE OLD.

The question of novelty in professional methods is a constant cause of friction. The young practitioner of a decade or two and the young graduate are practically upon the same level of thought, their minds apparently revolve in a circle of small diameter in which the present seems to be above and beyond anything heretofore attained, or that may be attainable in the future. This is not a pleasant or a profitable condition. It seems to be forgotten that progress is ever working spirally, always returning in part to a former position, and that, after all, the real advance made is not by any means so great as the surface indications would warrant us in believing. The dental periodical literature teems with the new-old things, and this is not always confined to methods and appliances, but occasionally some one will grow irritated because the older practitioners are unwilling to accept everything proposed as original with this generation. In illustration of this the following quotation from a discussion reported in a Western journal sufficiently illustrates. The speaker is made to say, "Every once in a while I hear some old fossil get up and say that in 1843, or '39, or '27, or something like that, all of these things were talked of and used for this, that, and the other purpose. But when you read the books that contain the description of the methods and treatment you will find that that was not true." While the expression "old fossil" seems to lack a reverential awe of the ancients in the dental profession, the subjects of this criticism have no reason to complain of this American way of treating old age, for it is felt to be peculiar to the rough and ready methods of thought and expression belonging to very recent civilizations. The old fossils are, however, not so far wrong as the critic would have us believe. Dental progress has been marked by successive steps, and it is not surprising that succeeding travellers tread irreverently in the footsteps of generations of workers in the same direction. The class who are constantly discovering processes in vogue fifty or more years ago are numerous. If, then, some one of the ancients rises to state this fact, he is met with the charge, "There is nothing in dental literature to substantiate your statement." Both parties to this conflict of opinion may have expressed a truth. Thirty, forty, fifty years ago dental journals were few. Those that existed did so under the greatest difficulties. Dentists would not write directly for the then existing periodicals, and the latter were poor in quantity of matter and poorer in quality. Dentists had not then recovered from the effect of generations of prejudice. They were in the intermediate period when the closed door in dentistry was slowly giving way to the open one. The man in practice was yielding unwillingly the secrets of his laboratory and operating-room. The multiplication of colleges and societies changed all this, but it has taken many years to accomplish it; indeed, it may be questioned whether we are as free to impart to-day as we would like to have each other believe.

Those who attended dental colleges fifty years ago received more than those not thus favored, and many things were taught there that were not generally known, and are frequently introduced now as absolutely new; and probably they are to the man of another generation.

Very recently we have been favored with lengthy dissertations on the filling of teeth. These are all interesting and valuable to the practitioner, but the new in them is principally based on the dictum of Dr. Black, published in 1891, that in the preparation of cavities "extension for prevention" must be the rule. This article led to the adoption of methods of operative procedures that have had their chief centre in a Western city. The writer has no criticism to make on this method of practice, preferring to let time demonstrate its value. Within limitations it is true, and it was considered so true fifty years ago that the operators of that period held it to be their duty to cut away on all proximate surfaces until they could reach solid structure. Extension for prevention is, therefore, by no means a modern thought. Indeed, some who had the pleasure of listening to Professor Elisha Townsend will recall the fact that he delineated the preparation of the "compound cavity," as he termed it,—the approximate and occlusal surfaces,—in almost similar terms to the "step" described by recent writers. It was not, however, illustrated in the books and dental periodicals of the period. The only really new thing in filling, outside of the appliances introduced, is the contouring of fillings up to the contact point, or, as Webb called it, "knuckling" the gold. This new method came in with cohesive gold, but it had a long struggle with earlier prejudices, and even now some doubt its value, contending, with some degree of force, that fewer teeth are saved by this process to-day than were saved under the old processes with non-cohesive gold.

The truth or falsity of this position is not of special interest at this writing. It seems, however, that in losing sight of old procedures and adopting the so-called new, the professional mind is giving up much of value and frequently choosing methods that may lead to serious results.

A recent writer says, "One other point in cavity building has been gaining ground slowly but surely. It has been called the "convenience form," or the cutting away of tooth-structure until we get an outline of the cavity that makes it easy of access. Indeed, cavities should be so constructed that all the walls are easily approached. All honor to the older operators who strove so hard to conserve tooth-structure, retain frail walls, and construct peculiarly shaped pluggers, that these cavities might be filled with some degree of perfectness. . . . Now we seek to prepare cavities easy of access with all points conveniently reached." This sounds very like the instruction received by the students of dentistry in 1854.

While this quotation seems to be in exact accord with the past, the author of it probably placed upon it another meaning; neither does extension for prevention mean exactly that which was held to be true under the non-cohesive method of filling teeth. While it combines the practice of a former period in cutting down frail walls, and also cutting for space, it extends this to the removal of sound tissue to meet possible future ravages of decay. This may be illustrated by extending a small cavity on the proximate surface of an incisor to and above the gingival border and the cutting away of the occlusal surface of a molar whether decay exists on that surface or not. It seems to the writer that this proceeding will not stand the test of time.

With all due respect to the ideas thus outlined, and at the risk of being considered a fossil, the writer feels that to make this method a success means wide separations. If these are made, as formerly, by the chisel there could be no criticism, for to attempt to fill a tooth properly without access to all portions of the cavity is simply an impossibility. While this is generally recognized as true, the modern operator proceeds by a combination of methods that seem to the writer to have in them an element of danger. Separations must be made, but how? The operator at the close of the nineteenth century says, "Place in between the teeth a separator and use force until sufficient room has been obtained." He fails to tell us what the effect of that force may be, and is generally quite indifferent as to pathological results. He should know that to secure space sufficient on proximate surfaces of teeth will require a movement of at least two millimetres. Consider for a moment what this means. The pericementum yields but slightly to pressure, and any increase of force must necessarily drive the alveolar process out of its natural position. The tendency of teeth is to move in the direction of the force applied, and will continue to move in the line of least resistance until a positive irregularity supervenes. This is not ordinarily applicable to the posterior teeth, but is a serious matter with the incisors, especially the laterals. It seems also to have been forgotten that the *extension* of the separation may mean extension of the inflammation produced by the force applied and the possibility of a resulting necrosis of the alveolar plate in anæmic subjects. The separator has a limit of usefulness, as well as the chisel; but the latter properly and intelligently used can do better and quicker work and with less pain to the patient than any separator, whether that be by screw force, cotton, gutta-percha, or wedges.

The writer previously quoted pats the ancients on the back figuratively and literally when he writes, "All honor to the older operators who strove so hard to *conserve tooth-structure, retain*

*frail walls,* and construct peculiarly shaped pluggers." This would be quite important information if it were true. The "older operators" did exactly the opposite, and cut and filed to an extent that would have horrified the conservator of tissue of the present period. The workers towards the close of the first half of the nineteenth century thoroughly believed in the extension of a cavity to render its filling not only easy but possible, and to prevent future decay,—extension for prevention. Then came a period succeeding this when conservatism prevailed, and this led to the use of the violent force to which allusion has been made.

The object of this article is not to treat this subject to the extent it deserves, but to call attention to the tendency to inaccurate statements and wrong thinking. The necessity for closer study of the past history of dentistry is every day becoming more and more apparent. It is unfortunate that the real record of facts is not always obtainable, hence the statements of the fossils alluded to should be received not only with respect, but as a means of accurately determining the status of the dental profession at a period antedating the memory of those actively engaged in the work.

Dentistry in its upward march differs in no respect from all other efforts among civilized peoples, a step forward and a step backward, but each step should and generally does mean improvement, the old becoming the new and the new returning in part to the old and again advancing spirally to that perfection which should be the aim of all truly professional men.

